# Threonyl-tRNA Synthetase Promotes T Helper Type 1 Cell Responses by Inducing Dendritic Cell Maturation and IL-12 Production *via* an NF-κB Pathway

**DOI:** 10.3389/fimmu.2020.571959

**Published:** 2020-10-14

**Authors:** Hak-Jun Jung, Su-Ho Park, Kyung-Min Cho, Kwang Il Jung, Daeho Cho, Tae Sung Kim

**Affiliations:** ^1^Department of Life Science, College of Life Science and Biotechnology, Korea University, Seoul, South Korea; ^2^Institute of Convergence Science, Korea University, Seoul, South Korea

**Keywords:** aminoacyl-tRNA synthetase, threonyl-tRNA synthetase, dendritic cell, interleukin-12, type 1 helper T cells, interferon-γ, influenza A virus

## Abstract

Threonyl-tRNA synthetase (TRS) is an aminoacyl-tRNA synthetase that catalyzes the aminoacylation of tRNA by transferring threonine. In addition to an essential role in translation, TRS was extracellularly detected in autoimmune diseases and also exhibited pro-angiogenetic activity. TRS is reported to be secreted into the extracellular space when vascular endothelial cells encounter tumor necrosis factor-α. As T helper (Th) type 1 response and IFN-γ levels are associated with autoimmunity and angiogenesis, in this study, we investigated the effects of TRS on dendritic cell (DC) activation and CD4 T cell polarization. TRS-treated DCs exhibited up-regulated expression of activation-related cell-surface molecules, including CD40, CD80, CD86, and MHC class II. Treatment of DCs with TRS resulted in a significant increase of IL-12 production. TRS triggered nuclear translocation of the NF-κB p65 subunit along with the degradation of IκB proteins and the phosphorylation of MAPKs in DCs. Additionally, MAPK inhibitors markedly recovered the degradation of IκB proteins and the increased IL-12 production in TRS-treated DCs, suggesting the involvement of MAPKs as the upstream regulators of NF-κB in TRS-induced DC maturation and activation. Importantly, TRS-stimulated DCs significantly increased the populations of IFN-γ^+^CD4 T cells, and the levels of IFN-γ when co-cultured with CD4^+^ T cells. The addition of a neutralizing anti-IL-12 mAb to the cell cultures of TRS-treated DCs and CD4^+^ T cells resulted in decreased IFN-γ production, indicating that TRS-stimulated DCs may enhance the Th1 response through DC-derived IL-12. Injection of OT-II mice with OVA-pulsed, TRS-treated DCs also enhanced Ag-specific Th1 responses *in vivo*. Importantly, injection with TRS-treated DC exhibited increased populations of IFN-γ^+^-CD4^+^ and -CD8^+^ T cells as well as secretion level of IFN-γ, resulting in viral clearance and increased survival periods in mice infected with influenza A virus (IAV), as the Th1 response is associated with the enhanced cellular immunity, including anti-viral activity. Taken together, these results indicate that TRS promotes the maturation and activation of DCs, DC-mediated Th1 responses, and anti-viral effect on IAV infection.

## Introduction

Aminoacyl-tRNA synthetases (aaRSs) are enzymes related to aminoacylation that cognate amino acid to a specific-tRNA. The aaRSs constitute 20 differential types, each transferring a specific amino acid. In addition to the essential function catalyzing aminoacylation function, some of the aaRSs are secreted into the extracellular environment, similar to cytokines, in specific conditions, and have various non-canonical functions. These include the control of transcription, translation (1) and RNA processing (2), as well as anti-viral immunity (3, 4), angiogenesis (1) tumorigenesis (5), metastasis (6), apoptosis, and immune response (6–8) including pro-inflammatory effect (9, 10).

Threonyl-tRNA synthetase (TRS) is a class II aaRS that charges threonine to threonine-specific tRNA. TRS has been associated with autoimmune diseases, such as polymyositis and dermatomyositis, as demonstrated by the presence of the myositis autoantibody PL-7 (11, 12). TRS is secreted into the extracellular space when vascular endothelial cells are exposed to tumor necrosis factor-α (TNF-α) or vascular endothelial growth factor (VEGF) associated with an angiogenetic environment. Secreted TRS can induce endothelial cell migration and angiogenesis (13) as well as pancreatic cancer cell migration (14). However, the effect of TRS on immunity involving dendritic cells (DCs) and DC-mediated T cell response remains unknown.

Dendritic cell is a type of professional antigen-presenting cells (APCs) that link innate and adaptive immunity. Activated and matured DCs exhibit characteristic phenotypic changes including the upregulation of the expression of MHC II and costimulatory molecules, such as CD40, CD80 and CD86. Moreover, mature DCs can secret various cytokines and chemokines (15) Naïve CD4 T cells can be differentiated through direct interaction with mature DCs, as well as by indirect communication by secreted various cytokines from DCs (16, 17). In addition, matured DCs induce the polarization of CD4^+^ T cell to T helper (Th) subsets, such as Th1, Th2, Th17 and regulatory T cell (T_reg_), under each polarizing condition containing specific cytokines (15) Th1 cells can be differentiated by interleukin (IL)-12 from DCs, and activated Th1 cells secrete a large amount of interferon-γ (IFN-γ) which is related in the upregulation of the cellular immune response and the clearance of pathogens, such as virus and bacteria (18, 19). Th2 cells produce IL-4, IL-5 and IL-13, which are critical for many diseases including rhinitis, asthma and atopic dermatitis (20). IL-17-secreting Th17 cells induce immune responses against self-antigens and autoimmune disease such as rheumatoid arthritis and systemic lupus erythematosus (21). In contrast, Foxp3-positive T_reg_ cells are directly involved in the regulation of immune response to pathogens, allergens, and self-antigens by tolerance and downregulation of effector T cells (22).

IFN-γ is the type II of IFNs that have essential role in combating virus infection and regulating the antiviral immune response (23). IFN-γ induces the antiviral immunity *via* regulation of the innate immune response and activation of adaptive immunity (18) against Ebola virus (24), Hepatitis B virus (25), Dengue virus (26), and Influenza A virus (IAV) (19).

Here, we report novel non-canonical functions of TRS whereby it induces the maturation and activation of DCs with Th1-polarizing ability and anti-viral activity. TRS induced the activation and maturation of bone marrow-derived DCs, as well as primary splenic DCs. TRS-activated DCs promoted Th1 responses *in vitro* and *in vivo*. Importantly, administration of TRS-stimulated DCs upregulated the populations of IFN-γ-secreting CD8^+^ and CD4^+^ T cells as well as secretion level of IFN-γ and induced viral clearance in influenza-infected mice. These results may explain the role of TRS in autoimmunity and angiogenesis, as well as its anti-viral activity.

## Materials and Methods

### Mice

Female C57BL/6N mice at 7–10 weeks of age were purchased from Orient Bio (Kapyong, Korea) and ovalbumin (OVA)-specific OT-II transgenic mice on a C57BL/6 background were purchased from Jackson Laboratory (Bar Harbor, Maine, USA). All mice were housed under specific pathogen-free conditions. All animal experiments were conducted according to the Korea University Guidelines for the Care and Use of Laboratory Animals (approval No. KUIACUC-2019-0013).

### Reagents and Cytokines

Cells were cultured in RPMI 1640 medium (Thermo Scientific, Rockford, IL) with 10% FBS (Capricon Scientific, Germany), 100 U/ml penicillin, 100 μg/ml streptomycin, 10 mM HEPES (Life Technologies BRL, Rockville, MD, USA), and 50 μM 2- mercaptoethanol (Sigma-Aldrich, St Louis, MO). Recombinant mouse GM-CSF was obtained from ProSpec (Rehovort, Israel). Recombinant human Flt3L was obtained from Biolegend (California, USA). Recombinant TRS was overexpressed as a His tag fusion protein in IPTG-induced *Escherichia coli* BL21 (DE3) and purified by nickel affinity chromatography, followed by a HiTrap Q column (GE Healthcare, 17-5156-01) for anion exchange chromatography. The eluent was further purified by gel filtration chromatography using Superdex75 16/600 (GE Healthcare, 28-9893-33) to further remove residual LPS. The level of endotoxin in each purification lot was determined using a Toxinsensor™ chromogenic LAL endotoxin assay kit (Genscript, Nanjing, China). Lots containing < 0.05 EU/μg protein were used for this study. Anti-phospho-ERK, anti-ERK, anti-p38, anti-phospho-JNK, anti-JNK, anti-IκBα, anti-IκBβ, anti-NF-κBp65 and anti-GAPDH Abs were from Santa Cruz Biotechnology (Dallas, TX). Anti-phospho-p38 Abs and anti-phospho-NF-κBp65 was purchased from Cell Signaling Technology (Beverly, MA). Anti-6X His tag Abs was purchased from Abcam (Cambridge, UK). Alexa Fluor 488-labeled anti-rabbit IgG and Alexa Fluor 488-labeled anti-mouse IgG were purchased from Molecular Probes (Eugene, OR). ERK inhibitor (U0126) was purchased from AbMole BioScience. NF-κB inhibitor (CAPE), IKK/IκB inhibitor (IKK-16), p38 inhibitor (SB203580), JNK inhibitor (SP 600125), OVA, LPS (from E. coli 0111:B4), PMA, and ionomycin were purchased from Sigma-Aldrich. Golgiplug containing Brefeldin A and anti-mouse IL-12p40/p70 (C17.8) were purchased from BD Biosciences (San Diego, CA).

### Generation of Bone Marrow-Derived DCs and Splenic DCs

The femurs and tibiae of C57BL/6 mice were cut and the marrows were flushed with ice-cold RPMI 1640 medium using syringe that was equipped with a 26-gauge needle. RBCs were lysed with RBC lysis buffer from Biovision (Milpitas, CA). The bone marrow cells were then suspended in growth medium. The number of cells was adjusted to 4 × 10^6^ cells/well (10 ml), and then added to petri dishes. The cells were cultured in RPMI 1640 medium containing 10% FBS, 100 U/ml penicillin, 100 μg/ml streptomycin, 10mM HEPES, and 50 μM β-mercaptoethanol supplemented with 20 ng/ml GM-CSF. The half of medium was renewed every other day, and the semi adherent cells were harvested on day 7 by gentle pipetting and used as immature GM-CSF-derived DCs. For Flt3L-derived DCs, BM cells were resuspended at 2 × 10^6^ cell/ml in RPMI 1640 medium containing 200 ng/ml human recombinant FMS-like tyrosine kinase 3 ligand (Flt3L, Biolegend, 550602), plated at 5 ml/well in 6 well plates and cultured for 9 days.

Splenic DCs were isolated from spleen cell suspensions using CD11c microbeads (CD11c MicroBeads UltraPure, Miltenyi Biotec) and MACS system (Miltenyi Biotec, San Diego, CA) with an MACS cell separator. CD11c^+^ splenic DCs from C57BL/6 mice (1.0 x 10^6^ cells/well) were cultured for 20 h in a 24-well plate with LPS (500 ng/ml) or TRS (200 nM). The cells were thoroughly washed and used for phenotypic and functional characterization by flow cytometric analysis and the culture supernatants were used for determining IL-12 levels by enzyme-linked immunosorbent assay (ELISA).

### Flow Cytometric Analysis

For cell surface molecules staining, DCs (1 x 10^6^ cells/well) were harvested, washed with FACS washing buffer (0.5% FBS and filtered 0.05% NaN_3_ in PBS). The cells were then labeled for 20 min at room temperature with APC-conjugated anti-mouse CD11c (N418) and one of the following PE-conjugated mAbs: anti-mouse IA^b^ (AF6-120.1; MHC II), anti-mouse CD40 (3/23), anti-mouse CD80 (16-10A1) and anti-mouse CD86 (GL1) The mean fluorescence intensity (MFI) of each surface molecules was measured from the total cell population *via* BD accuri C6 plus flow cytometer (BD Biosciences). For intracellular staining, cells were stimulated for 5 h with 50 ng/ml of PMA, 1 μg/ml of ionomycin and Golgiplug. The cells were harvested, washed with FACS buffer and stained with FITC-conjugated anti-mouse CD4 (RM4-5) for 20 min at room temperature. The cells were fixed for 20 min using a Cytocfix/Cytoperm kit (BD Biosciences). Intracellular cytokines were detected with APC-conjugated anti-mouse IFN-γ (XMG1.2) and IL-17 (eBio17B7), PE-conjugated anti-mouse IL-4 (11B11). Treg cells were firstly stained with FITC-conjugated anti-mouse CD4 (RM4-5) and PE-conjugated anti-mouse CD25 (PC61), followed by APC-conjugated anti-mouse FoxP3 (FJK-16s) according to the manufacturer’s instructions of FoxP3 staining buffer set (eBioscience, San Jose, CA).

### Reverse Transcription Polymerase Chain Reaction

Total mRNA was extracted from the DCs (3 x 10^6^ cells/well) by using RiboEX reagent (GeneAll Biotechnology, Korea) according to the manufacturer’s protocol. Total RNA was reverse transcribed into cDNA with CycleScript reverse transcriptase (Bioneer) and amplified by PCR. After PCR amplification, the products were separated on 1.5% agarose gels and stained with ethidium bromide. Primer sequences used in this study are: mouse *IL-1β* (forward, 5′-CTGAAGCAGCTATGGCAACT-3′; reverse, 5′-ACAGGACAGGTATAGATTC-3′), *IL-6* (forward, 5′-TGAACAACGATGATGCACTT-3′; reverse, 5′-CGTAGAGAACAACATAAGTC-3′), *IL-12p35* (forward, 5′-TCAGCGTTCCAACAGCCTC-3′; reverse, 5′-TAAAACGCAGCTCAG TAACAGTCCG-3′), *IL-12p40* (forward, 5′-CAGAAGCTAACCATCTCCTGGTTTG-3′; reverse, 5′-TCCGGAGTAATTTGGTGCTTCACAC-3′), *TNF-α* (forward, 5′-GGCAGGTCTACTTTGG AGTCATTG-3′; reverse, 5′-ACATTCGAGGCTCCAGTGAATTTCGG-3′), *TGF-β* (forward, 5′-TATAGCAACAATTCCTGGCG T-3′; reverse, 5′-TCCTAAAGTCAATGTACAGCT-3′), *GAPDH* (forward, 5’-ACATCAAGAAGGTGGTGAAG- 3’; reverse, 5’-ATTCAAGAGAGTAGGGAGGG-3’).

### Enzyme-Linked Immunosorbent Assay

The concentrations of IL-12p40, IL-12p70, IL-4, and IL-17 in the culture supernatants were measured in triplicate using ELISA kit (eBioscience, San Diego, CA). The level of IFN-γ was quantified with specific murine anti-IFN-γ HB170 coating and biotinylated anti-IFN-γ XMG1.2 mAbs and the standard curve was generated using recombinant IFN-γ (BD Bioscience). The wells were finally washed again with PBST (0.05% Tween-20 in PBS) and *o*-phenylenediamine (OPD) containing citrate and H_2_O_2_ was added to each well and incubated for 20 min at room temperature. To stop the reaction, 2 N H_2_SO_4_ was added to each well. Developed colors were detected on a VMax kinetic microplate reader at 490 nm. The measurement was performed in triplicate.

### Immunofluorescent Staining

DCs (3 x 10^5^ cells/well) were fixed with 4% paraformaldehyde and blocked with 0.1% Triton X-100 and 0.5% BSA in PBS for 30 min at RT. The cells were incubated with rabbit anti-NF-κBp65 (1:300 dilution) and mouse Anti-6X His tag Abs (1:300 dilution), at 4°C overnight, followed by staining with Alexa Fluor 488–conjugated anti-rabbit IgG Ab (1:300 dilution) and Alexa Fluor 488–conjugated anti-mouse IgG Ab (1:300 dilution) for 1 h at RT. Actin were counterstained with Rhodamine phalloidin (Molecular Probes, 1:200 dilution) for 1 h at RT. Nuclei were counterstained with DAPI (Molecular Probes, 3 μM) for 3 min and observed with a confocal laser scanning microscope (LSM 800, Carl Zeiss, Oberkochen, Germany).

### Western Blot Analysis

DCs (5 x 10^6^ cells/well) were harvested at the indicated time points, washed with PBS, and lysed in RIPA buffer containing protease and phosphatase inhibitor cocktail. The whole cell lysates were then separated on 10% SDS-PAGE and transferred to nitrocellulose membranes. The membranes were blocked with 4.5% BSA for 1 h, and incubated overnight at 4°C with respective Abs against phosphorylated p38, JNK, ERK, IκBα, IκBβ, NF-κBp65 or GAPDH. The membranes were then treated with HRP-conjugated anti-mouse IgG or anti-rabbit IgG at room temperature and the bands were visualized with chemiluminescent HRP substrate (Millipore Corporation, Billerica, MA) and X-Ray film processor (JP-33, JPI, Seoul, Korea).

### CD4^+^ T Cell Isolation and Th Subtype Polarization *In Vitro* and *In Vivo*

CD4^+^ T cells were isolated from lymph nodes of mice by magnetic bead purification (MACS, Miltenyi Biotec.), and purity was > 95%. For *in vitro* experiments of Th cell polarization, purified CD4^+^ T cells (1 x 10^5^ cells) were incubated for 2 or 3 days with anti-CD3 Ab and anti-CD28 Ab (1 μg/ml) in the presence of specific polarizing cytokines of each Th subset as follows: IL-12p70 (10 ng/ml) and anti-IL-4 (20 ng/ml) for Th1, IL-4 (20 ng/ml) and anti-IFN-γ (20 ng/ml) for Th2; TGF-β (2 ng/ml), IL-6 (20 ng/ml), anti-IL-4 (20 ng/ml) and anti-IFN-γ (20 ng/ml) for Th17; TGF-β (2 ng/ml) for Treg polarization. For the *in vitro* syngeneic co-culture of CD4^+^ T cells and DCs, isolated CD4^+^ T cells (5 x 10^5^ cells) from lymph node of OT-II mice were mixed with OVA-pulsed immature DCs, or OVA-pulsed LPS- or TRS-treated DCs (5 x 10^4^ cells) at a ratio of 10:1. Three days later, the cell was harvested for analyzing populations of Th subsets, and the supernatant was collected for determining levels of IFN-γ, IL-4, and IL-17. The 7-day cultured DCs from C57BL/6 mice were pulsed for 2 h with 10 ng/ml OVA, after which the cells were incubated for 6 h with LPS (500 ng/ml) or TRS (50, 100, and 200 nM). For *in vivo* polarization, OVA-pulsed, media-DCs, or TRS-treated DCs (1 x 10^6^ cells) were adoptively transferred into OT- II mice by subcutaneous injection *via* footpads. Seven days later, CD4^+^ T cells were collected form the draining lymph nodes and cultured in the presence of OVA (100 ng/ml).

### Immunization and Influenza Virus Infection

Female 7-weeks-old C57BL/6 mice were immunized with DCs. The 7-day cultured immature DCs from C57BL/6 mice were pulsed for 3 h with 10 μg/ml hemagglutinin (HA) of influenza A/WSN/1933 (Sino Bio, China), after which the cells were incubated for 24 h in the presence or absence of TRS (200 nM). One day later, the cell was harvested and HA-pulsed, media-incubated DCs, or HA-pulsed, TRS-treated DCs (1 x 10^6^ cells) were injected into the mouse tail *via* intravenously (i.v.) injection. A negative control (MOCK) were also included. Three days after DC injection, mice were infected intranasally (i.n.) with 1 mLD_50_ (mouse-lethal dose) of H1N1 IAV in 100 μl. H1N1 IAV were provided by Prof. Sang-Yun Choi (27, 28). All procedures were performed under isoflurane anesthesia. Virus infected mice were observed and weighed daily for 7 days. Total weight loss of > 30% were considered as humane endpoint.

### Plaque Assay

Virus titration in the lung supernatants were detected by the standard plaque assay. The Monolayer Madin-Darby canine kidney (MDCK) cells were infected with 10-fold serially diluted lung supernatant for 1 h in Opti-MEM (Invitrogen) supplemented with 0.3% bovine serum albumin (BSA), 0.5 μg/ml TPCK-treated trypsin and 1% antibiotics. After virus infection, the media were replaced with overlay media (L15 media containing 0.3% BSA, 1 μg/ml TPCK-treated trypsin, 1% antibiotics, 1% glutamine, 2% high malting agarose). After incubation for 72 h, MDCK cells were fixed with 4% formaldehyde for 1 h. The overlay agarose was removed by flowing water and stained with 0.2% crystal violet solution. Viral titer were determined by counting the number of plaque.

### Analysis of Cell Populations in Bronchoalveolar Lavage Fluid

Mice were anesthetized and bronchoalveolar lavage fluid (BALF) was collected by 1 ml of PBS twice through the trachea. The BALF cells and lung lymphocytes were harvested, washed with FACS buffer and stained with FITC-conjugated anti-mouse CD4 (RM4-5) and CD8 (53-6.7) for 20 min at room temperature. The cells were fixed for 20 min using a Cytocfix/Cytoperm kit (BD Biosciences). Intracellular cytokines were detected with APC-conjugated anti-mouse IFN-γ (XMG1.2). The stained cells were analyzed by using FACS Accuri with Accuri C6 software (BD Biosciences).

### Statistical Analysis

Statistical analysis was performed with unpaired Student’s t-test for pairwise comparisons in SigmaPlot version 12.5 software (Systat Software Inc. Washington, USA) or one-way analysis of variance (ANOVA) with a Bonferroni post-test for multiple comparisons and using Kaplan-Meier method and a log-rank test (survival) and Mann-Whitney U test (body weight) in IBM SPSS Statistic 25 software (IBM. NY, USA). A *P*-values less than 0.05 were considered to be statistically significant. The data were presented as the means ± SD or SEM of three independent experiments.

## Results

### TRS Induced the Activation and Maturation of Bone Marrow-Derived DCs

**Figure 1:**
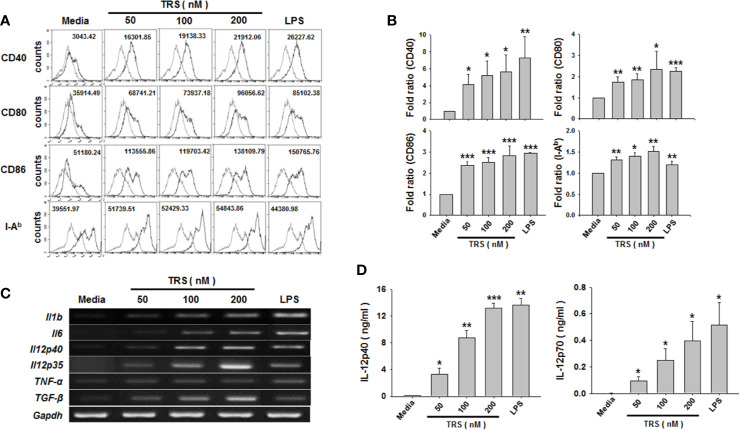
Threonyl-tRNA synthetase (TRS) increases the expression levels of surface molecules and cytokines in bone marrow-derived dendritic cells (DCs). Bone marrow-derived DCs were isolated from C57BL/6 mice as described in the Materials and Methods. Immature-DCs (iDCs) were cultured for 20 h **(A, D)**, or 6 h **(C)** with threonyl-tRNA synthetase (TRS; 50, 100, and 200 nM) or lipopolysaccharide (500 ng/ml). **(A)** The expression of CD40, CD80, CD86, and I-A^b^ molecules, as detected by flow cytometry for CD11c^+^ gated cells. Gray histograms, isotype control; black histograms, anti-CD40, anti-CD80, anti-CD86, and anti-I-A^b^ Ab. Representative figures are presented. The value shown in the histograms represents the mean fluorescence intensity. **(B)** The fold ratio of surface molecules expression was plotted with the media-treated DCs as 1.0. The data represent mean ± SD of three independent experiments (n = 3); *P < 0.05, **P < 0.01 and ***P < 0.001 compared with media-treated DCs. **(C)** mRNA levels of pro-inflammatory cytokines, as confirmed by reverse transcriptase-polymerase chain reaction. GAPDH is used as a control. **(D)** Levels of IL-12p40 and IL-12p70 in cell supernatants, as detected by enzyme-linked immunosorbent assay. Experiments were conducted three times independently and are represented as the mean ± SEM of results performed in triplicate (n = 3). Statistical significance was assessed using unpaired Student’s *t*-test; *P < 0.05, **P < 0.01 and ***P < 0.001 compared with media-treated DCs.

When immature DCs are matured, they have the ability to activate and polarize CD4^+^ T cells *via* several signals, such as costimulatory molecules, MHC class molecules, and cytokines. To investigate whether TRS stimulated the expression of these maturation-related cell surface molecules on DCs, bone marrow-derived DCs were incubated for 20 h in the presence or absence of TRS and the expression of the cell surface molecules was assessed by cytofluorometric analysis. TRS significantly increased the expression of MHC II and the costimulatory molecules CD40, CD80 and CD86 on CD11c^+^ DCs in a dose-dependent manner, as observed by the mean fluorescence intensity (MFI) values *via* cell cytometry. (**Figures 1A, B**).

To exclude the possibility of endotoxin contamination in TRS-mediated expression of cell surface molecules, DCs were pretreated with polymyxin B (PMB) to inhibit LPS signaling, followed by incubation with TRS or LPS. Treatment of DCs with PMB significantly inhibited the LPS-induced increase of the expression levels of all three surface molecules. However, PMB treatment did not affect the TRS-mediated expression of the cell surface molecules (**Supplemental Figure 1A**). In addition, the expression of cell surface molecules remained unchanged, similar to the case for the media-treated DCs, if the TRS protein was boiled for 30 min at 100°C before the treatment of the DCs (**Supplemental Figure 1B**), indicating that the TRS-mediated increase in the expression of cell surface molecules was not due to LPS contamination. Moreover, less than 0.05 endotoxin unit/mg of protein (1 endotoxin unit = 0.1 ng/ml *E. coli* LPS) was detected, as determined by the Limulus Amebocyte lysate assay. These endotoxin concentrations proved insufficient for the activation of DCs.

Maturation and activation of DCs affect the expression of several genes, including cytokine genes. To investigate whether TRS stimulated the production of Th cell-polarizing cytokines from DCs, DCs were treated with TRS and the expression of the cytokines were determined at the mRNA and protein levels by reverse transcription polymerase chain reaction (RT-PCR) and ELISA. The mRNA levels of IL-1β, IL-6, TNF-α, TGF-β, IL-12p35, and IL-12p40 were significantly increased in TRS-treated DCs a dose-dependent manner. (**Figure 1C** and **Supplemental Figure 2**). Consistent with mRNA levels, TRS significantly enhanced secretion of IL-12p40 and IL-12p70 in a dose-dependent manner (**Figure 1D**).

In general, classical mouse DCs is known to express integrin α X chain protein CD11c, and they have been identified as plasmacytoid DC (pDC) and conventional DC (cDC). Moreover, cDC can be further divided into the related lymphoid resident CD8^+^XCR1^+^ DC (cDC1) and CD8^-^CD103^+^ DC (cDC2) (29, 30). According to recently reported papers, BMDCs are generated by two strategies using GM-CSF or Flt3L and they constituted of distinctly different DC subsets. Flt3L-derived DC is defined as a steady state DC, which contains pDC and two cDC subsets. However, GM-CSF-derived DC is defined as an inflammatory DC and does not include the cDC1 (31, 32). Therefore, we confirmed the effect of TRS on DC maturation by using BMDCs generated through Flt3L treatment. As shown, TRS significantly increased the expression of MHCII and co-stimulatory molecules, including CD40, CD80 and CD86, on Flt3L-derived DCs in a dose-dependent manner (**Supplemental Figure 3A, B**). In addition, TRS significantly increased the secretion levels of IL-12p40 in a dose-dependent manner (**Supplemental Figure 3C**). Taken together, these results indicate that TRS promoted the maturation and activation of both GM-CSF-derived DCs and Flt3L-derived DCs.

### The MAPK and NF-κB Signaling Pathways Were Involved in TRS-Induced Activation of DCs

**Figure 2:**
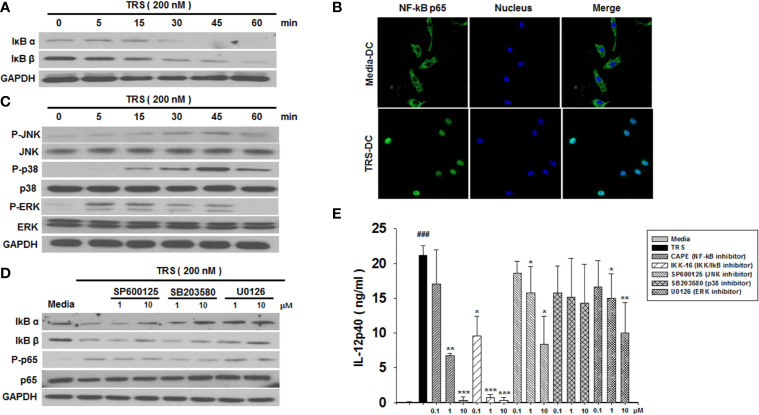
Threonyl-tRNA synthetase (TRS) activates dendritic cells (DCs) through the NF-κB and MAPK signaling pathways. **(A)** IκBα, IκBβ, and GAPDH expression levels, as measured by western blotting of DCs treated with TRS in a time-dependent manner (0–60 min). **(B)** The nuclear translocation of NF-κB p65, as detected by confocal laser scanning microscopy (LSM 800, Carl Zeiss, Oberkochen, Germany) of DCs treated with TRS (200 nM) for 1 h and then incubated with an anti-NF-κB p65 antibody (Ab), followed by staining with an Alexa Fluor 488-conjugated Ab and 4′,6-diamidino-2-phenylindole (DAPI). **(C)** DC culture method described in **(A)**. Western blotting for phosphorylated JNK, p38, and ERK or unphosphorylated JNK, p38, and ERK. **(D)** Levels of IκBα, IκBβ, NF-κB p65, and phosphorylated NF-κB p65, as detected *via* western blot analysis of DCs pretreated with the MAPK inhibitors (1, 10 μM) and treated for 30 min with TRS. **(E)** IL-12p40 levels in the supernatant, as detected *via* ELISA of DCs pretreated with the indicated inhibitors (0.1, 1, 10 μM) for 30 min and treated with TRS for 20 h. The media-treated DCs without inhibitors was analyzed as a control. Experiments were conducted three times independently and are represented as the mean ± SEM of results performed in triplicate (n = 3). Statistical significance was assessed using unpaired Student’s *t*-test; ^###^*P* < 0.001 compared with media-treated DCs, *P < 0.05, **P < 0.01 and ***P < 0.001 as determined by one-way analysis of variance with a Bonferroni post-test for multiple comparisons.

The observation that TRS-treated DCs induced the maturation and production of IL-12 led us to investigate the underlying immune signaling pathways. The NF-κB and MAPK signaling pathways are known to play an essential role in DC maturation and cytokine production. As shown in **Figure 2A**, TRS-treated DCs exhibit the degradation of IκBα and IκBβ proteins in a time-dependent manner. In addition, the nuclear translocation of NF-κB p65 following TRS treatment was detected *via* immunofluorescence (**Figure 2B**). To gain further insights into TRS-induced signaling, we determined the phosphorylation of c-Jun N-terminal kinase (JNK), p38, and extracellular-signal-regulated kinase (ERK) in TRS-treated DCs. TRS induced the phosphorylation of JNK, p38, and ERK in a time-dependent manner (**Figure 2C**). Furthermore, to determine the relationship between NF-κB and MAPKs, we investigated the potential effect of TRS-induced NF-κB activation of DCs in the presence of JNK, p38 and ERK inhibitors. The treatment with MAPK inhibitors significantly decreased the degradation of IκBα and IκBβ proteins and reduced the phosphorylation of NF-κB p65 in TRS-treated DCs (**Figure 2D** and **Supplemental Figure 4**), suggesting that MAPKs were involved in the upstream signaling required for TRS-induced NF-κB activation. TRS-mediated IL-12 production was abolished by treatment of DCs with inhibitors of IκB kinase (IKK), NF-κB p65, JNK, and ERK in a dose-dependent manner. In contrast, the p38 kinase inhibitor had no effect (**Figure 2E**). Taken together, our results indicated that TRS induced the maturation and activation of DCs through the MAPK/NF-κB pathways.

The initiation of biological responses by signaling molecules, including endogenous proteins and hormones is induced by their interaction with cell surface molecules. To investigate whether TRS could attach to the surface of DCs, DCs were incubated with TRS and the localization of TRS was observed by immunofluorescence. TRS was observed to localize on the surface of DCs (**Supplemental Figure 5**).

### TRS Enhanced Th1 Responses *via* DC-Derived IL-12

**Figure 3:**
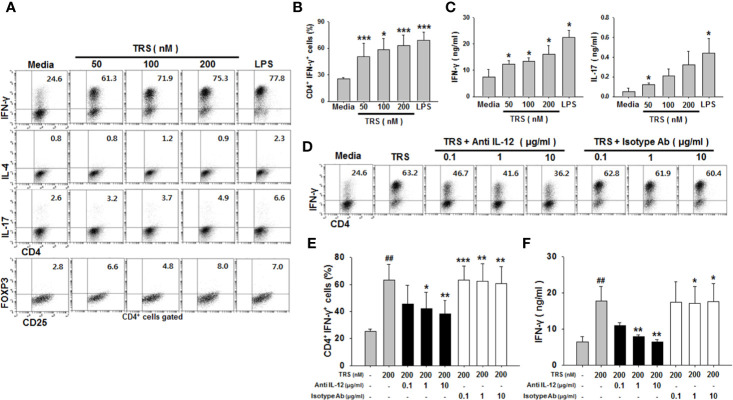
Threonyl-tRNA synthetase (TRS) induces the polarization of Th1 cells through the secretion of IL-12 from DCs. Dendritic cells (DCs) were treated with ovalbumin (OVA, 10 μg/ml) for 2 h and OVA-DCs were treated with TRS (50, 100, and 200 nM) or LPS (500 ng/ml) for 6 h. DCs were cultured with OT-II CD4^+^ T cells at a 1:10 ratio for three days. **(A)** IFN-γ-, IL-4-, IL-17- and Foxp3-expressing CD4^+^ T cell populations, as analyzed by flow cytometry. Representative figures are presented. **(B)** The data are expressed as the mean ± SD of three independent experiments (n = 3). **(C)** Protein levels of IFN-γ and IL-17 in the supernatant, as analyzed using ELISA. Data shown represent the mean ± SEM of three independent experiments (n = 3). **(B, C)** *P < 0.05, and ***P < 0.001 compared with media-treated DCs. **(D)** IFN-γ^+^ CD4^+^ T cells, as analyzed *via* flow cytometry, of DCs treated with OVA (10 μg/ml) for 2 h and then treated with 200 nM TRS for 6 h. The DCs were cultured with OT-II CD4^+^ T cells at a 1:10 ratio in the presence of anti-IL-12 antibody (Ab) (0.1–1 μg/ml) or isotype Ab (0.1–1 μg/ml) for three days. Representative figures are presented. **(E)** The population of IFN-γ^+^CD4^+^ T cells is presented as the mean ± SD of three independent experiments (n = 3). **(F)** Protein levels of IFN-γ in the supernatants, as detected by ELISA. Data shown represent the mean ± SEM of three independent experiments (n = 3). **(E, F)** Statistical significance was assessed using unpaired Student’s t-test; ^##^*P* < 0.01 compared with CD4^+^ T cells co-cultured with media-treated DCs, *P < 0.05, **P < 0.01 and ***P < 0.001 as determined by one-way analysis of variance with a Bonferroni post-test for multiple comparisons.

Mature DCs are able to induce the polarization of CD4^+^ T cells into subsets, including Th1, Th2, Th17, and T_reg_ cells, depending on the cytokines they produce. We demonstrated that TRS significantly increased the production of IL-12, a strong Th1-polarizing cytokine. Therefore, to investigate whether TRS-stimulated DCs polarized naïve CD4^+^ T cells into Th1 cells, ovalbumin (OVA)-pulsed DCs were treated with TRS and then co-cultured with naïve OT-II CD4^+^ T cells, followed by the assessment of Th subset populations by cytofluorometric analysis. As shown in **Figures 3A, B**, TRS-treated DCs significantly increased the percentage of the IFN-γ^+^CD4^+^ T cells (Th1) in a dose-dependent manner. However, TRS-treated DCs did not affect the populations of IL-4^+^CD4^+^ T cells (Th2) and FoxP3^+^CD4^+^ T cells (T_reg_). TRS-treated DCs was also likely to increase the population of IL-17^+^CD4^+^ T cells (Th17), although not statistically significant. TRS-treated DCs significantly increased IFN-γ production of co-cultured CD4^+^ T cells in a dose-dependent manner, although TRS partly promoted IL-17 secretion in the co-cultured CD4^+^ T cells (**Figure 3C**). To confirm whether TRS directly affected the polarization of CD4^+^ T cells without DCs, naïve CD4^+^ T cells were incubated in the presence or absence of TRS under the polarizing conditions of each Th subset, and the levels of each Th subset population were assessed. TRS did not directly affect the polarization of CD4^+^ T cells (**Supplemental Figure 6**).

Th1 cell polarization and IFN-γ production in CD4^+^ cells are known to correlate with IL-12, a Th1-polarizing cytokine. To investigate whether IL-12 secretion from TRS-stimulated DCs was related to the increased Th1 cell population and IFN-γ production, neutralizing anti-IL-12 monoclonal antibodies (mAbs) were added to the co-cultures of TRS-stimulated DCs and CD4^+^ T cells, and the levels of Th1 cell populations and IFN-γ production were assessed. The addition of anti-IL-12-neutralizing mAbs significantly decreased the polarization of CD4^+^ IFN-γ^+^ cells (**Figures 3D, E**), as well as IFN-γ production from CD4^+^ T cells (**Figure 3F**). However, the IgG isotype control antibody had no effect on the Th1 response. Taken together, these results showed that TRS induced the polarization of naïve CD4^+^ T cells into Th1 cells *via* DC-derived IL-12.

### TRS Stimulated the Maturation of Splenic DCs and TRS-Treated Splenic DCs Induced Th1 Polarization

**Figure 4:**
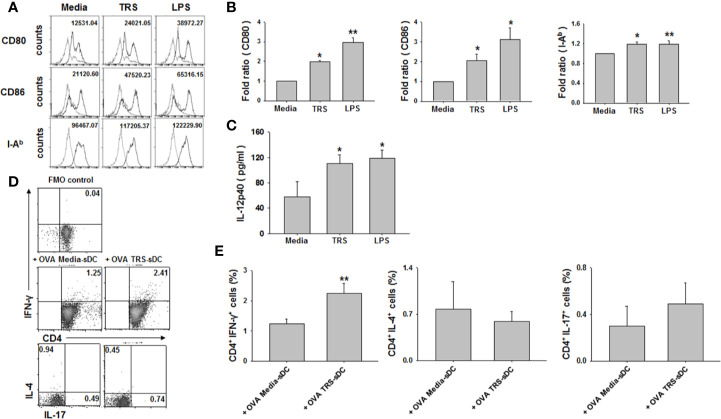
Threonyl-tRNA synthetase (TRS) induces the maturation of primary splenic DCs and promotes the polarization of Th1 cells. Splenic dendritic cells (DCs) were isolated from mouse spleen using CD11c MicroBeads (Miltenyi Biotec) and primary splenic DCs were cultured for 20 h with TRS (200 nM) or LPS (500 ng/ml). **(A)** The expression of CD80, CD86, and I-A^b^ molecules, as detected by flow cytometry for CD11c^+^ gated cells. Gray histograms, isotype control; black histograms, anti-CD80, anti-CD86, and anti-I-A^b^ Ab. Representative figures are presented. The value shown in the histograms represents the mean fluorescence intensity. **(B)** The fold ratio of surface molecules expression was plotted with the media-treated DCs as 1.0. The data represent mean ± SD of three independent experiments (n = 3); *P < 0.05 and **P < 0.01 compared with media-treated DCs. **(C)** Levels of IL-12p40 in cell supernatants, as detected by enzyme-linked immunosorbent assay (ELISA). Experiments were conducted three times independently and are represented as the mean ± SEM (n = 3). Statistical significance was assessed using unpaired Student’s *t*-test; *P < 0.05 compared with media-treated DCs. **(D)** IFN-γ-, IL-4-, and IL-17-expressing cells, as detected by flow cytometry for CD4^+^ gated cells of splenic DCs treated with OVA (10 μg/ml) for 2 h and then treated with media or TRS (200 nM) for 6 h. The DCs were cultured with OT-II CD4^+^ T cells at a 1:5 ratio for three days. Some cells were only stained with CD4 to generate FMO control for IFN-γ. Representative figures are presented. **(E)** The populations of CD4^+^-IFN-γ^+^, -IL-4^+^, and -IL-17^+^ cells are presented as the mean ± SD of three independent experiments (n = 3). *P < 0.05 and **P < 0.01 compared with the group injected with OVA, media-treated DCs.

We further examined the effect of TRS on DC maturation and the Th1 response using primary splenic DCs. Isolated primary DCs from mouse spleen were used to detect the levels of maturation in the following conditions: media treatment, TRS treatment, and LPS treatment. TRS-treated splenic DCs increased the expression of CD80, CD86, and MHC class II molecules (**Figures 4A, B**). Consistent with the analysis of BMDCs, secretion of IL-12p40 was promoted by TRS treatment in splenic DCs (**Figure 4C**). In addition, TRS-activated splenic DCs selectively increased the polarization of IFN-γ^+^CD4^+^ T cells from the OT-II naïve CD4^+^ T cells. IL-17^+^CD4^+^ T cell population also increased, although not statistically significant (**Figures 4D, E**). These results confirmed that TRS induced the maturation of not only bone marrow-derived DCs, but also that of primary splenic DCs.

### TRS-Treated DCs Induced a Th1 Response *In Vivo*

**Figure 5:**
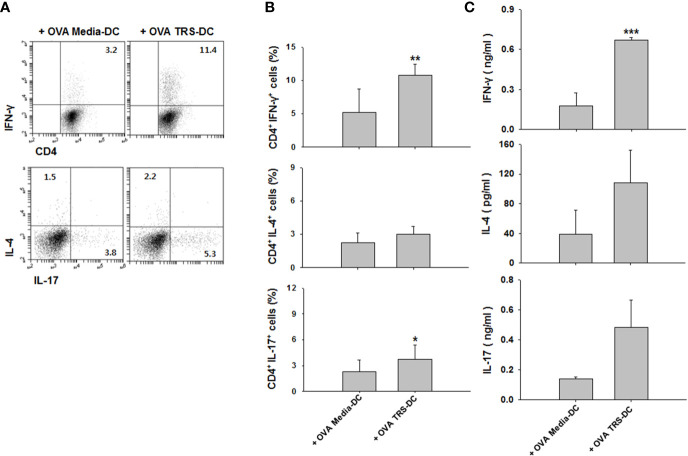
Threonyl-tRNA synthetase (TRS)-treated DCs induce Th1 polarization *in vivo*. Dendritic cells (DCs) were isolated from C57BL/6 mice as described in the Materials and Methods. Immature-DCs (iDCs) were cultured with ovalbumin (OVA, 10 μg/ml) for 2 h and OVA-DCs were treated with media or TRS (200 nM) for 6 h, harvested, and then, DCs were subcutaneously injected into OT-II mice through the footpad. After seven days, the draining lymph node cells were isolated and cultured for three days in the presence with OVA (100 μg/ml). **(A)** The populations of IFN-γ-, IL-4-, and IL-17-expressing CD4^+^ T cells, as analyzed by flow cytometry. Representative figures are presented. Results from **(A)** are summarized in **(B)** as the mean ± SD of three independent experiments (n = 6). **(C)** Protein levels of IFN-γ, IL-4, and IL-17 in the culture supernatant, as analyzed using enzyme-linked immunosorbent assay. Data shown represent the mean ± SEM of three independent experiments (n = 6). **(B, C)** *P < 0.05, **P < 0.01, ^##^*P* < 0.01, and ***P < 0.001 compared with the group injected with OVA, media-treated DCs.

To confirm the *in vivo* relevance of our observations, OVA-pulsed, TRS-treated DCs were adoptively transferred to OT-II mice. Seven days later, Th1, Th2 and Th17 cytokine profiles were measured in the presence of OVA in the draining lymph nodes of DC-immunized mice. Consistent with the results from *in vitro* co-cultures, immunization of mice with TRS-stimulated DCs significantly upregulated the populations of IFN-γ^+^CD4^+^ T cells and IL-17^+^CD4^+^ T cells, compared with the media-treated-DC-immunized mice. The population of IL-4^+^CD4^+^ T cells showed similar levels in both media-treated DC- and TRS-treated DC-immunized mice (**Figures 5A, B**). Furthermore, immunization of mice with TRS-stimulated DCs significantly increased IFN-γ production. However, levels of IL-4 exhibited no differences in both the media-treated DC and TRS-treated DC groups. The secretion of IL-17 was likely to show an increased pattern by TRS-stimulated DCs, but has no statistical significance (**Figure 5C**). These results demonstrated that TRS significantly enhanced a Th1 response *in vivo*.

### TRS-Treated DCs Showed an Increased Anti-Viral Effect Against IAV *In Vivo*

**Figure 6:**
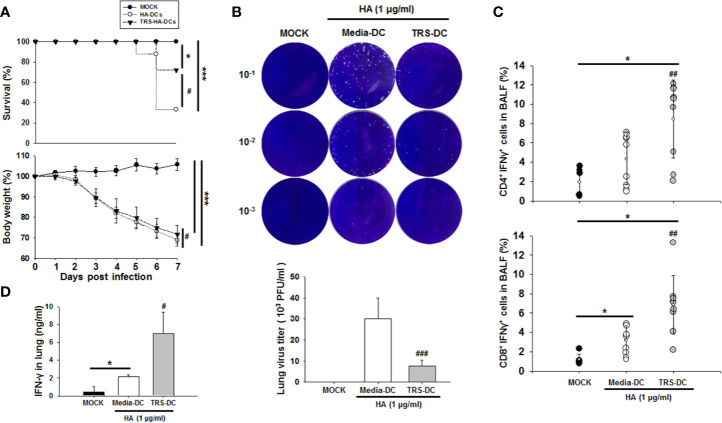
Threonyl-tRNA synthetase (TRS)-treated DCs induce an anti-viral effect in influenza virus A/WSN/1933 (H1N1)-infected mice. Female 7-week-old C57BL/6 mice were immunized with hemagglutinin (HA)- or TRS-HA-DCs. Three days later, mice were infected intranasally with 1 mLD_50_ of H1N1 influenza. **(A)** After infection for 7 days, infected mice were monitored for body weight loss and survival. Data represent n= 12 mice (MOCK), n=18 mice (HA-DC), and n=18 mice (TRS-HA-DC) from three independent experiments. Data shown are the mean ± SD and statistical significance was analyzed using Kaplan-Meier method and a log-rank test (survival) and Mann-Whitney U test (body weight); *P < 0.05, and ***P < 0.001 compared with the group injected with PBS-injected groups (MOCK), ^#^*P* < 0.05 compared with HA-DCs-injected groups. **(B)** Plaque assays of live influenza from whole lung at 7 days after infection. **(C)** Quantification of IFN-γ^+^CD4^+^ T cells and IFN-γ^+^CD8^+^ T cells identified in the bronchoalveolar lavage fluid by flow cytometry. Data represent n= 6 mice (MOCK), n=9 mice (HA-DC), and n=9 mice (TRS-HA-DC) from three independent experiments. **(D)** Protein levels of IFN-γ in the lung, as detected by ELISA Data shown represent the mean ± SEM of results performed in triplicate. Data shown are the mean ± SD **(B, C)**, ± SEM **(D)** and statistical significance was assessed using unpaired Student’s *t*-test; *P < 0.05 compared with the group injected with PBS-injected groups (MOCK), ^#^*P* < 0.05, ^##^*P* < 0.01, and ^###^*P* < 0.001 as determined by one-way analysis of variance with Bonferroni post-test for multiple comparisons.

IFN-γ is a type II interferon and has been reported to have an anti-viral effect similar to that of type I interferon (19), and it is known that the increase in the population of antigen (Ag)-specific Th1 cells affects the CD8^+^ T cell population (3). To investigate whether the TRS-DC-mediated-Th1 response had an anti-IAV effect, hemagglutinin (HA)-pulsed, TRS-DCs were used to immunize mice. HA is a well-known model Ag of IAV and has been used to stimulate an IAV-specific immune response (33, 34). Recombinant HA protein had no effect on DC maturation and activation (data not shown). As shown in **Figure 6A**, TRS-DC-immunized mice exhibit a higher survival ratio and body weight compared with media-treated DC-immunized mice. Moreover, TRS-DC-immunized mice significantly induced IAV clearance in lungs compared with the media-treated DC-immunized mice (**Figure 6B**). The populations of IFN-γ^+^CD4^+^ T cells and IFN-γ^+^CD8^+^ T cells were significantly upregulated in bronchoalveolar lavage fluid (BALF) of TRS-DC-immunized mice (**Figure 6C**). In addition, IFN-γ levels were higher in the lungs of TRS-DC-immunized mice, compared to those in the media-treated DC-immunized mice (**Figure 6D**). Taken together, these data revealed that TRS had an anti-IAV effect through the DC-mediated increase in IFN-γ-secreting CD4^+^ and -CD8^+^ T cell populations.

## Discussion

The aaRSs, secreted from several different types of cells, including cancer and immune cells, have a variety of extracellular functions (5, 13, 35–37). TRS, a class II aaRS, is secreted into the extracellular space upon exposure to TNF-α or VEGF from vascular endothelial cells (13). As TRS is also found in the sera of autoimmune patients, it is of interest to investigate its non-translation functions, especially, its cytokine-like activity in the regulation of inflammatory responses and the immune modulatory functions of DCs, which shape Ag-specific T cell immunity. In the present study, the data suggested that TRS was a novel effector that promoted the phenotypic and functional maturation and activation of DCs. TRS significantly upregulated the expression levels of costimulatory and MHC class II molecules on DCs. In addition, TRS increased the production of pro-inflammatory cytokines including IL-12 in DCs. Moreover, the IL-12 secreted from TRS-stimulated DCs triggered the polarization of CD4^+^ T cells to a Th1 subset. This is the first study to demonstrate that TRS may exhibit cytokine-like functions to induce the maturation and activation of DCs, and also to promote a Th1 response in a DC-dependent manner. This finding may also explain the role of TRS in autoimmune diseases and angiogenesis associated with the Th1 subset and IFN-γ.

Most of our *in vitro* experiments were conducted using bone marrow-derived DCs, which is an inflammation-specific DC. It is unclear whether these results are similar in different types of DCs, such as monocyte-derived DCs and tissue-resident DCs. In this study, we also included primary splenic DCs to confirm the TRS-induced DC-activation and Th1 polarization. As monocyte-derived DCs and tissue-resident DCs, including splenic DCs, are major populations in the total DC population (29–32), we expect that TRS is an effective molecule for DC activation and maturation.

TRS-mediated induction of Th1 response may occur through DC-derived IL-12. IL-12 is an important cytokine for the polarization of IFN-γ-secreting CD4^+^ T cells (15). TRS-treated DCs upregulated the secretion of IL-12 and increased the population of IFN-γ-secreting CD4^+^ T cells in co-cultures of DCs and CD4^+^ T cells. The addition of a neutralizing anti-IL-12 mAb to the co-culture system significantly suppressed the population of IFN-γ-secreting CD4^+^ T cells and IFN-γ secretion. In the co-cultured of TRS-DCs and CD4^+^ T cells in the presence of anti-IL-12 Ab, we confirmed that IL-12 was neutralized by the anti-IL-12 Ab addition in a dose-dependent manner. (**Supplemental Figure 7A**), clearly indicating that the Th1 polarization in the co-culture system was mainly due to the secreted IL-12 from TRS-DCs. Furthermore, in addition to IL-12, other factors may be additionally involved in the Th1 responses increased by TRS-DCs. The anti-IL-12 mAb addition did not completely neutralize the Th1 responses induced by TRS-DCs, although it decreased the levels of Th1 responses (IFN-γ-secreting CD4^+^ T cell population and IFN-γ secretion) to the basal levels, similar to those in the co-culture of media-DCs and CD4 T cells. TRS treatment increased co-stimulatory molecules (CD40, CD80, and CD86, **Figures 1A, B**) and Th1-related cytokines in DCs (IL-1β, IL-6 and TNF-α, **Figure 1C** and Supplement Figure. 2).

Moreover, injection of OT-II mice with TRS-treated, OVA-pulsed DCs increased the OVA-specific Th1 cell population. In contrast, TRS did not affect the polarization of other CD4^+^ T cell subsets, such as Th2, Th17, and T_reg_ cells in co-cultures of DCs and CD4^+^ T cells (**Figure 3**). Furthermore, TRS did not directly induce CD4^+^ T cell polarization in the presence of CD3 and CD28 stimuli (**Supplemental Figure 6**), indicating that TRS increased Th1 responses *via* IL-12 secreted from DCs matured and activated by TRS treatment.

TRS-treated DCs partially increased the secretion of IL-17 (**Figures 3C** and **4D, E**) and population of IL-17+ CD4+ cells in the co-culture system and *in vivo* experiments (**Figures 5A, B**), although the increased levels were not statistically significant. Therefore, it is likely that TRS partially affected the polarization of Th17 cells and IL-17 secreted from Th17 cell. In addition, Th1 cell population may be a source of the increased IL-17 as Th1 cells are reported to secrete IL-17 in a pro-inflammatory environment (**Supplemental Figure 7B**) (38). Further research is needed to investigate the direct effect of TRS on Th17 responses and any contribution of Th17 cells in Th1 responses.

TRS-mediated induction of IL-12 production in DCs may occur *via* NF-κB activation through MAPK signaling, involving the upstream molecules of NF-κB. DC maturation *via* the MAPK and NF-κB signaling pathways is well known (34–41). MAPK and NF-κB are conserved signaling components that play essential roles in both innate and adaptive immune responses. Three major molecules involved in MAPK signaling are: ERK, p38 MAP kinase, and JNK (40). Moreover, the intracellular signaling pathways, including NF-κB, are associated with the regulation of pro-inflammatory cytokines following the stimulation of macrophages and DCs (41, 42). We have shown that TRS significantly induced the maturation of DCs through phosphorylation of JNK, and ERK, and activation of the NF-κB pathways. In addition, we confirmed decreased IL-12 secretion following treatment with JNK and ERK inhibitors. Although the p38 inhibitor suppressed the activation of NF-kB signaling pathways in the TRS-treated DCs, it failed to block the secretion of IL-12p40. Consequently, TRS induced DC maturation and secretion of IL-12 *via* the ERK/JNK-mediated NF-κB pathways.

Exogenous molecules that cannot pass through the cell membrane must directly interact with the cell surface in order to induce the biological functions of target cells. Although a receptor for TRS has not been reported, our data raises the question that TRS is probably related to the surface of DC and could initiate a signaling cascade for DC maturation and activation. We confirmed the localization of TRS on the surface of DCs by immunofluorescent microscopic analysis, although not sufficient to prove the direct interaction (**Supplemental Figure 5**). Cell surface receptors for other aaRSs have been reported, although further studies are needed to define a receptor of TRS on APCs, as well as the effect of TRS on other APCs. Lysyl-tRNA synthetase (KRS) interacts with the 67-kDa laminin receptor (67LR), leading to cancer cell migration during metastasis (43, 44).

IFN-γ is an anti-viral cytokine, although relatively weak compared with IFN-α or IFN–β (18, 19, 24, 25). In addition, DCs are important sensors to detect viral products and alert the immune system to the presence of virus (45, 46). We demonstrated that TRS increased IFN-γ secretion through a DC-mediated Th1 response. Therefore, we conducted experiments to confirm the anti-viral effect of TRS in mice infected with IAV. HA-pulsed, TRS-treated DCs, instead of the whole TRS protein, were used for immunization to exclude the effects of natural killer cells or other APCs, including macrophages and B cells. In addition, HA is well-known as a surface Ag of A/WSN/1933 (H1N1) influenza virus and is used for Ag-specific immune responses (33, 34). HA itself, did not affect the activation and maturation of DCs (data not shown). Adoptive transfer of HA-pulsed, TRS-treated DCs significantly increased the populations of IFN-γ^+^CD4^+^ T cells and IFN-γ^+^CD8^+^ T cells in the BALF and decreased the number of live influenza particles in the lung, resulting in an increased survival period and bodyweight of influenza-infected mice.

In addition, we investigated the effects of various recombinant aa-tRNA synthetases and their complexed components (AIMP2, DRS, HRS, LRS, TRS, KRS, and YRS) on DC, and only KRS and TRS induced maturation and activation of DCs with different extents (data not shown). Although TRS and KRS are categorized into the same group in aaRS classification, this is just a functional classification. In addition, TRS and KRS induced DC maturation through distinctly different pathways ERK phosphorylation is a representative difference occurring only in TRS-, not KRS-induced DC maturation.

In summary, we demonstrated that TRS enhanced a Th1 response through the maturation and activation of DCs with Th1-polarizing ability, which was mainly due to the increased IL-12 production by the MAPK/NF-κB pathways. These data may explain the presence of autoantibodies in patients with autoimmune diseases, and also suggest TRS as a novel candidate for a protective adjuvant against respiratory viruses, including IAV.

## Data Availability Statement

The raw data supporting the conclusions of this article will be made available by the authors, without undue reservation.

## Ethics Statement

The animal study was reviewed and approved by Institutional Animal Care and Use Committee of Korea University.

## Author Contributions

H-JJ designed and performed all the experiments. H-JJ, S-HP, K-MC, DC, and TK analyzed and interpreted the experimental results. KIJ provided the IAV and protocol for influenza infection in mouse model. DC contributed reagents/materials. H-JJ and TK collaborated on manuscript writing. TK supervised the study and corrected the manuscript. All authors contributed to the article and approved the submitted version.

## Funding

This work was supported by the National Research Foundation of Korea (NRF) grant (NRF-2017R1A2B2009442) and the Korea University grant.

## Conflict of Interest

The authors declare that the research was conducted in the absence of any commercial or financial relationships that could be construed as a potential conflict of interest.

## Abbreviations

aaRS, aminoacyl-tRNA synthetase; DC, dendritic cell; IAV, influenza A virus; IFN-γ, interferon-gamma; LPS, lipopolysaccharide; MFI, mean fluorescence intensity; OVA, ovalbumin; PMB, polymyxin B; TRS, threonyl-tRNA synthetase.
