# Supplementary material for: Threonyl-tRNA Synthetase Promotes T Helper Type 1 Cell Responses by Inducing Dendritic Cell Maturation and IL-12 Production via an NF-κB Pathway
Source: Front Immunol. 2020 Oct 14;11:571959. doi: 10.3389/fimmu.2020.571959 (PMC7592646; doi:10.3389/fimmu.2020.571959)
Supplement: Supplementary file 1 [file DataSheet_1.pdf]

Supplemental data

Threonyl-tRNA synthetase promotes T helper type 1 cell responses by inducing dendritic cell maturation and IL-12 production via an NF-κB pathway

Hak-Jun Jung[1], Su-Ho Park[1], Kyung-Min Cho[1], Kwang Il Jung[1], Daeho Cho[2,3], and Tae Sung Kim[1*]

[1]Department of Life Science, College of Life Science and Biotechnology, Korea University, Seoul 02841, Republic of Korea
[2] Institute of Convergence Science, Korea University, Seoul 02841, Republic of Korea
[3]Kine Sciences, 525 Seolleung-ro, Gangnam-gu, Seoul 06149, Republic of Korea

*** Correspondence:**
Tae Sung Kim
tskim@korea.ac.kr

Keywords: aminoacyl-tRNA synthetase$_1$, threonyl-tRNA synthetase$_2$, dendritic cells$_3$, interleukin-12$_4$, type 1 helper T cells$_5$, interferon-γ$_6$, influenza A virus$_7$.

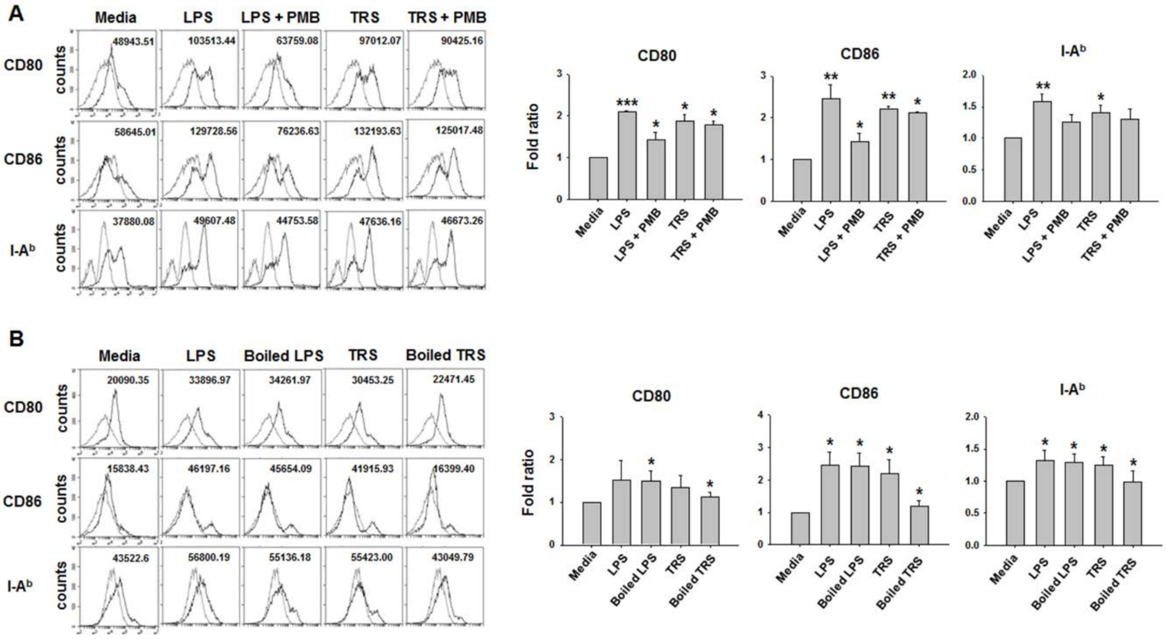

Supplemental Figure 1. TRS-induced maturation of DCs is not due to LPS contamination. (A) DCs were pretreated with PMB (20 µg/ml) for 30 min and then cultured with TRS (200 nM) or LPS (500 ng/ml) for 20 h. The expression levels of CD80, CD86 and I-A^b molecules were detected by flow cytometry for CD11c$^+$ gated cells. Gray histogram, isotype control; black histograms, anti-CD80, anti-CD86, anti-I-A^b Ab, respectively. Representative figures are presented. (B) DCs were treated with TRS (200 nM), LPS (500 ng/ml) or boiled TRS (200 nM), boiled LPS (500 ng/ml) (boiled TRS and boiled LPS were incubated during the 5 min at 95℃ for inactivation of protein) for 20 h. The expression of CD80, CD86 and I-A^b molecules were detected by flow cytometry for CD11c$^+$ gated cells. Representative figures are presented. The value shown in the histograms represents the mean fluorescence intensity. (A, B) The fold ratio of surface molecule expression levels were plotted with the media-treated DCs as 1.0. The data were represented as means ± SD of three independent experiments (n = 3); * $P < 0.05$, ** $P < 0.01$ and *** $P < 0.001$ compared with media-treated DCs.

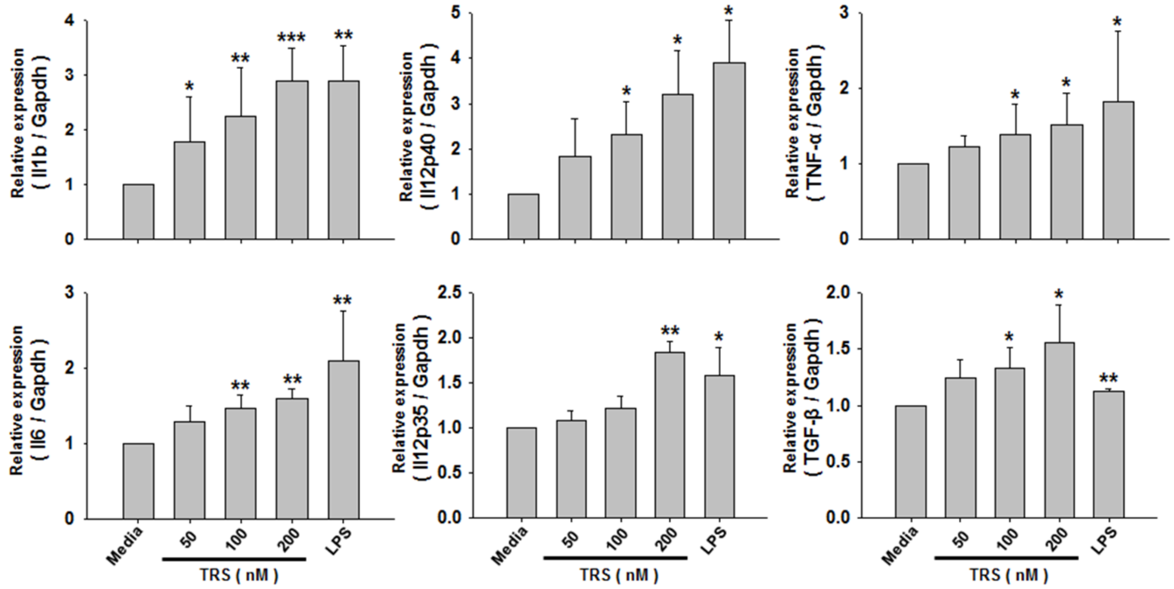

Supplemental Figure 2. TRS induces the production of pro-inflammatory cytokines from DCs.

In Fig. 1C, intensities of the bands were quantified by ImageJ (NIH, Wisconsin, USA) and the fold changes in gene expression compared to GAPDH were calculated, and the relative expressions are represented compared to the media-treated DCs as 1.0. The data represent mean ± SD of three independent experiments (n = 3); * $P < 0.05$ and ** $P < 0.01$ compared with media-treated DCs.

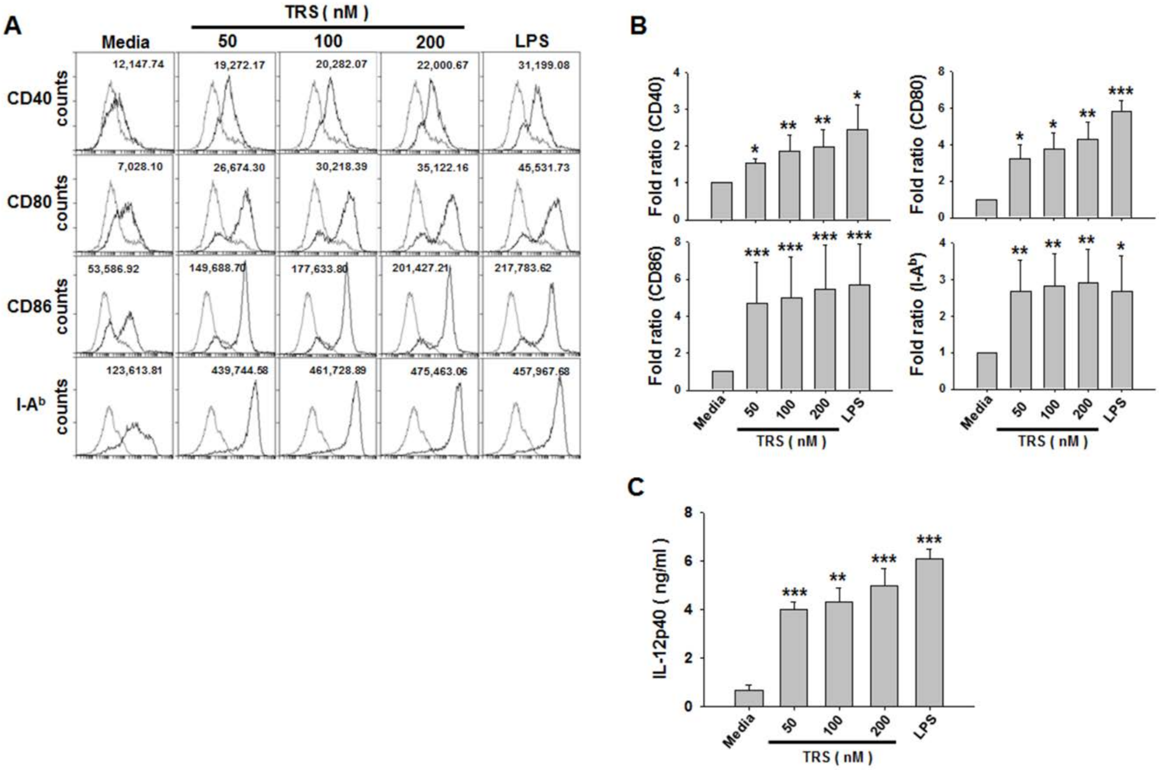

Supplemental Figure 3. TRS promotes the expression levels of cell surface markers and IL-12 in Flt3L derived DCs.

BMDCs were isolated from C57BL/6 mice and generated by recombinant Flt3L as described in the Materials and Methods. Harvested Flt3L-DCs were cultured for 24 h with TRS (50, 100 and 200 nM) or LPS (200 ng/ml). (A) The expression of CD40, CD80, CD86, and I-A^b molecules, as detected by flow cytometry for CD11c$^+$ gated cells. Gray histograms, isotype control; black histograms, anti-CD40, anti-CD80, anti-CD86, and anti-I-A^b Ab. Representative figures are presented. The values shown in the histograms represent the mean fluorescence intensity. (B) The fold ratio of surface molecules expression was plotted compared with the media-treated DCs as 1.0. The data represent mean ± SD of three independent experiments (n = 3); * $P < 0.05$, ** $P < 0.01$ and *** $P < 0.001$ compared with media-treated DCs. (C) Levels of IL-12p40 in cell supernatants, as detected by ELISA. Experiments were conducted three times independently and are represented as the mean ± SEM of results performed in triplicate (n = 3). Statistical significance was assessed using unpaired Student's *t*-test; ** $P < 0.01$ and *** $P < 0.001$ compared with media-treated DCs.

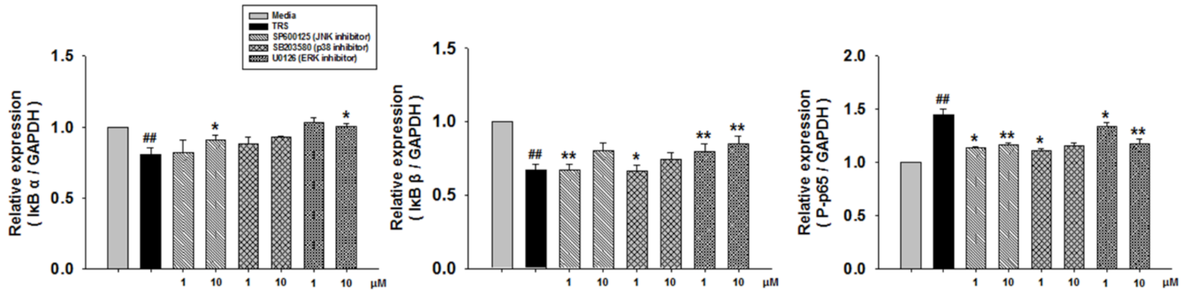

Supplemental Figure 4. MAPK inhibitors suppress the TRS-induced activation of NF-κB signaling pathway in DCs.

In Fig. 2D, intensities of the bands were quantified by ImageJ (NIH, Wisconsin, USA) and the fold changes in gene expression compared to GAPDH were calculated, and the relative expressions are represented compared to the media-treated DCs as 1.0. The data represent mean ± SD of three independent experiments (n = 3). Statistical significance was assessed using unpaired Student's *t*-test; ## $P < 0.01$ compared with media-treated DCs, * $P < 0.05$ and ** $P < 0.01$ as determined by one-way analysis of variance with a Bonferroni post-test for multiple comparisons.

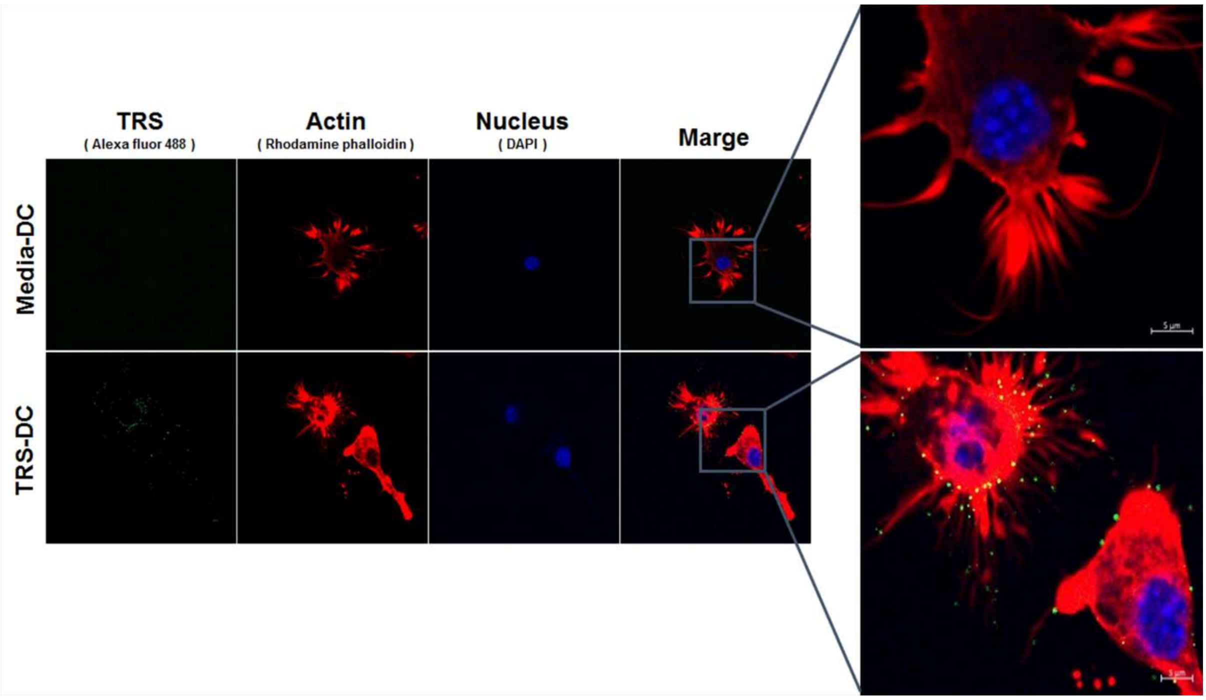

Supplemental Figure 5. TRS directly binds to the surface of DCs.

DCs were treated with TRS (300 nM) for 30 min and then the DCs were incubated with anti-6X His tag Abs, followed by staining with Alexa Fluor 488-conjugated Ab, rhodamine phalloidin (actin) and DAPI (nucleus). The 6X His tagged TRS shown as green dots was detected by confocal laser scanning microscope (LSM 800, Carl zeiss, Oberkochen, Germany). Scale bars, 5μm. Representative figures are presented.

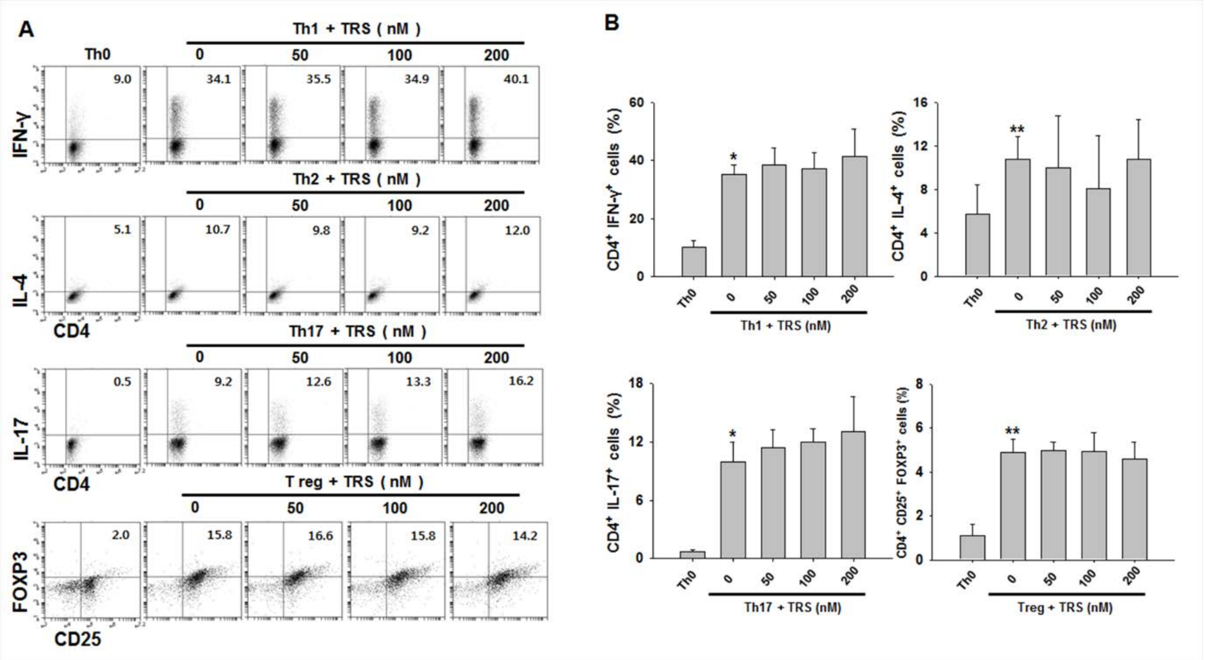

Supplemental Figure 6. TRS does not directly affect the polarization of Th cells.
The naïve CD4+ T cells were isolated and differentiated into the Th1-, Th2-, Th17-, and T_{reg} cells under each specific polarizing condition in the absence or presence of various concentrations of TRS (0, 50, 100, and 200 nM) with CD3 and CD28. (A) After 2 days (Th1 and Th17) or 3 days (Th2 and T_{reg}) of incubation, the CD4+, IFN-γ-, IL-4-, IL-17-, and FOXP3-T cells were detected using flow cytometry. Representative figures are presented. Results from (A) are summarized in (B) as mean ± SD of three independent experiments (n = 3). * $P < 0.05$ and ** $P < 0.01$ compared with a group of Th0 cells.

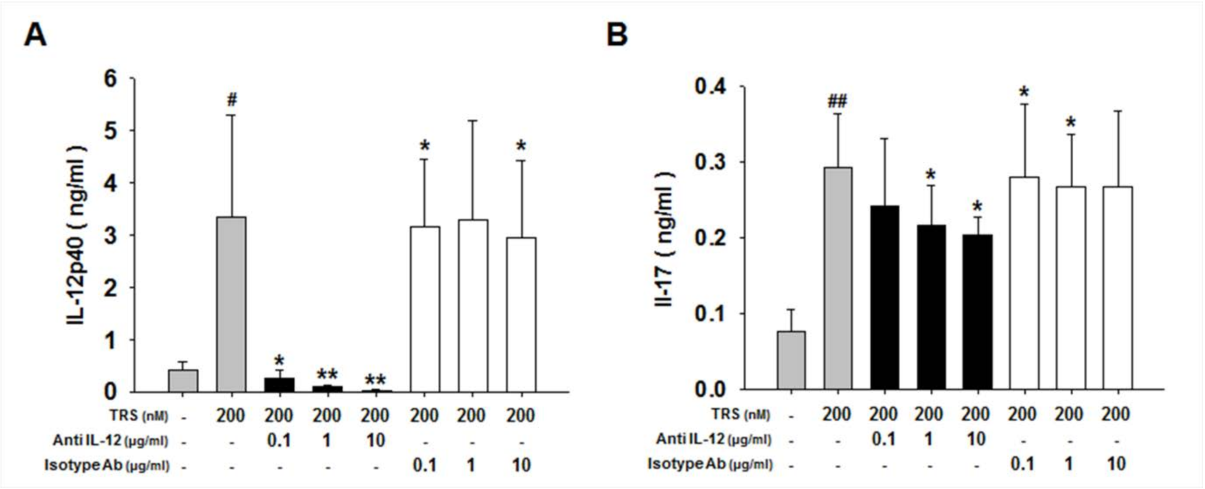

Supplemental Figure 7. TRS induced polarization of Th1 cells through the IL-12.
DCs treated with OVA (10 μg/mL) for 2 h and then treated with 200 nM TRS for 6 h. The DCs were cultured with OT-II $CD4^+$ T cells at a 1:10 ratio in the presence of anti-IL-12 antibody (Ab) (0.1-1 μg/mL) or isotype Ab (0.1-1 μg/mL) for three days (Fig. 3D). (A) Protein levels of IL-12p40 and (B) protein levels of Il-17 in the supernatants, as detected by ELISA. Data shown represent the mean ± SEM of three independent experiments (n = 3). Statistical significance was assessed using unpaired Student’s t-test; # $P < 0.05$ and ## $P < 0.01$ compared with $CD4^+$ T cells co-cultured with media-treated DCs, * $P < 0.05$ and ** $P < 0.01$ as determined by one-way analysis of variance with a Bonferroni post-test for multiple comparisons.
